# Final recommendations for the Latin America and Caribbean Periodontics Consensus 2024

**DOI:** 10.1590/1807-3107bor-2024.vol38.0122

**Published:** 2025-01-10

**Authors:** Cristina Cunha VILLAR, Paola CARVAJAL, Fernanda CARRER, Giuseppe Alexandre ROMITO, James Rudolph COLLINS, Marco Antonio ALARCÓN, Cassiano Kuchenbecker RÖSING, Juliano CAVAGNI, Andres Duque DUQUE, Gloria Inés LAFAURIE Villamil, Ricardo Guimarães FISCHER, Bernal STEWART, Zilson MALHEIROS, Carlos BENÍTEZ, Claudio Mendes PANNUTI

**Affiliations:** (a) Universidade de São Paulo – USP, School of Dentistry, Department of Periodontics, São Paulo, SP, Brazil.; (b) Universidad de Chile, School of Dentistry, Department of Conservative Dentistry, Santiago, Chile.; (c) Universidade de São Paulo – USP, School of Dentistry, Department of Community Dentistry, São Paulo, SP, Brazil.; (d) Pontificia Universidad Católica Madre y Maestra, School of Dentistry, Department of Periodontology, Santo Domingo, Republica Dominicana.; (e) Universidad Peruana Cayetano Heredia, Academic Department of Clinical Stomatology, Lima, Perú.; (f) Universidade Federal do Rio Grande do Sul – UFRGS, Faculty of Dentistry, Department of Periodontology, Porto Alegre, RS, Brazil.; (g) CES University, School of Dentistry, Department of Periodontics, Medellín, Colombia.; (h) Universidad El Bosque, School of Dentistry, Unit of Basic Oral Investigation, Bogotá, Colombia.; (i) Pontifícia Universidade Católica do Rio de Janeiro – PUCRS, School of Dentistry, Department of Periodontics, Rio de Janeiro, RJ, Brazil.; (j) Latin American Oral Health Association – LAOHA, São Paulo, SP, Brazil.; (k) Colgate-Palmolive Company, Colgate Technology Center, Piscataway, NJ, USA.

**Keywords:** Periodontal Diseases, Public Health, Latin America, Caribbean Region, Prevalence, Risk Factors, Diagnosis

## Abstract

The 2024 Consensus on Periodontology for Latin America and the Caribbean addresses the significant public health issue of periodontal diseases, impacting millions in the region. This comprehensive document presents holistic recommendations to standardize diagnostic methodologies, enhance public awareness, and integrate preventive and therapeutic practices into general healthcare. Key areas include understanding the prevalence and impact of periodontal diseases, identifying risk factors, and improving diagnostic, preventive, and treatment strategies. The consensus emphasizes interdisciplinary collaboration, tailored public health interventions, and the importance of continuous monitoring and research. By unifying efforts across various sectors, the consensus aims to reduce the burden of periodontal diseases, thereby improving both oral and general health outcomes in Latin America and the Caribbean.

## Introduction

The 2024 Consensus on Periodontology for Latin America and the Caribbean represents a landmark endeavor to systematically address periodontal disease, a significant public health issue impacting millions in the region. Developed by the concerted efforts of expert stakeholders, this consensus aims to unify and enhance periodontal health practices, research, and public awareness, by laying out a set of holistic recommendations. These guidelines advocate for standardized methodologies, increased public awareness, and the integration of diagnostic, preventive, and therapeutic practices into general healthcare, alongside a push for research and education to empower dental professionals. By addressing these areas in a focused and collaborative approach, the consensus aims to significantly reduce the burden of periodontal diseases, thereby improving both oral and general health across Latin America and the Caribbean. The following recommendations were established based on the content of the six consensus papers:^1-6^

## Prevalence of Periodontal Diseases

### Standardize examination methodologies

Advocate for the adoption of standardized methodologies and case definitions for periodontal disease across epidemiological research studies to ensure comparability and enhance the understanding of disease prevalence and trends. Develop and disseminate a training module for epidemiological surveys including periodontal disease assessment to achieve consistency in periodontal disease assessment across studies, observing WHO guidelines.^7^

### Establish a regional expert panel

Form a panel of regional experts tasked with the periodic review and update of case definitions and diagnostic criteria for periodontal diseases, ensuring alignment with global standards while accounting for regional variations.

### Implement a standardized surveillance framework

Introduce a standardized framework for periodontal disease surveillance that includes specific indicators for ongoing monitoring and evaluation efforts, with the aim of capturing accurate prevalence data.

### Promote multicenter studies and collaboration

Encourage the development of national oral health surveillance systems and foster international collaborations for multicenter studies. These initiatives will contribute to a deeper understanding of the epidemiology of periodontal diseases within the region.

### Create a regional consortium for periodontal research

Establish a consortium to support resource sharing, methodological alignment, and data pooling. Additionally, launch a database for periodontal health to facilitate collaboration and informed decision-making.

### Obtain support for research

Seek funding and logistical backing for multicenter studies by means of partnerships with global health organizations, government agencies, and the private sector, with the aim of bolstering research infrastructure for periodontal disease.

### Prioritize underrepresented populations in research

Increase research efforts targeting underrepresented groups, such as rural and indigenous communities, by using culturally sensitive methodologies to ensure accurate and comprehensive data collection. Develop programs to engage underrepresented populations in oral health research and interventions, thereby enhancing community participation and data collection efforts across diverse demographic groups.

### Address socioeconomic and educational determinants

Emphasize the need for strategies that address the link between periodontal disease prevalence, socioeconomic and educational factors. Advocate for policy reforms that integrate oral health into the broader primary healthcare system, thereby promoting equitable access to care.

### Enhance continuous monitoring and evaluation

Support the development of a regional oral health surveillance system to continuously monitor periodontal disease trends and the impact of public health interventions. Encourage the adoption of electronic health records and mobile health technologies for efficient data collection and analysis, with the aim of improving the overall management of, and prevention strategies for periodontal diseases.

## Burden and Impact of Periodontal Diseases on Oral Health-Related Quality of Life and Systemic Diseases

### Expand epidemiological research

Broaden the representation of epidemiological studies across LAC countries to better understand the relationship between periodontal diseases and Oral Health-Related Quality of Life (OHRQoL). These studies should focus on diverse cultural and socioeconomic contexts to provide a comprehensive view of periodontal health impacts.

### Conduct longitudinal studies on periodontal health and systemic diseases

Encourage multicenter longitudinal studies to be conducted to investigate the associations and potential causative links between periodontitis and non-communicable diseases (NCDs), such as cardiovascular diseases, diabetes, and, respiratory conditions.

### Address vulnerable populations

Direct research efforts towards assessing the impact of periodontal diseases on the quality of life among vulnerable groups, including those that are geographically isolated or with restricted access to dental care, those living in poverty and indigenous populations.

### Evaluate the cost-effectiveness of periodontal interventions

Conduct intervention studies to assess the effectiveness and cost-efficiency^8^ of periodontal treatments in managing or mitigating systemic conditions.

### Integrate oral health into general healthcare

Advocate for the integration of oral health assessments and periodontal disease management into the broader healthcare system. This includes training healthcare providers to recognize the systemic implications of periodontal health and ensuring that oral health is considered an integral part of overall health and well-being.

### Public health programs

Develop and disseminate targeted public health programs to raise awareness about the significant impact of periodontal diseases on quality of life and its association with systemic conditions.

## Risk Factors of Periodontal Disease

### Enhanced training on risk factors

Ensure that dental professionals, right from their undergraduate training onwards, receive proper training in identifying and managing key periodontal disease risk factors, with particular emphasis on smoking^9^ and diabetes.^10,11^ Dental education curricula should be updated to include in-depth coverage of risk factor management for periodontal disease. This includes training in conducting effective patient interviews and applying current evidence-based practices for risk factor modification.

### Public awareness programs and policies

Implement widespread public health programs to increase awareness of the significant impact of smoking and poorly managed diabetes on periodontal health. By leveraging various media platforms and targeting both the general public and high-risk groups, these programs can play a crucial role in promoting early intervention and encouraging healthier lifestyles.

### Integration of risk factor screening into dental evaluations

Advocate for the routine incorporation of risk factor screening for smoking and diabetes into dental evaluations, irrespective of the patient’s initial reason for consultation. This approach can facilitate prompt and effective preventive measures and treatments.

### Customize public health interventions

Develop and implement public health interventions that are specifically designed to reduce the prevalence of smoking and improve diabetes management in Latin American and Caribbean populations. Tailored approaches are necessary to address the unique challenges and circumstances of the region.

### Promote interdisciplinary collaboration

Foster interdisciplinary collaboration between dental professionals and other healthcare specialists, such as endocrinologists and tobacco cessation specialists, to ensure a comprehensive approach to managing periodontal disease risk factors.

### Longitudinal research

Encourage and support the conducting of longitudinal cohort studies within the region to increase elucidation of the causal relationships between smoking, diabetes, and periodontal disease.

## Enhancing Periodontal Disease Diagnosis

### Integrate training in diagnostics into dental education

Incorporate extensive training on periodontal disease diagnosis into dental education curricula, with emphasis on early detection and application of the AAP/EFP^12^ classification. This initiative aims to equip new dental professionals with the knowledge necessary for effective diagnosis from the outset of their careers.

### Launch public awareness programs

Execute targeted public awareness programs to raise knowledge about the signs and symptoms of periodontal diseases. Use culturally relevant media channels to ensure widespread outreach and impact, with the aim of empowering individuals with the knowledge to seek early treatment.

### Promote validated self-report tools

Advocate for the use of validated self-report tools in both clinical settings and public health initiatives. These tools are designed to help with early recognition of periodontal diseases by patients themselves, thereby facilitating timely professional consultation.

### Standardize full-mouth periodontal examinations

Push for the adoption of full-mouth periodontal examinations as the standard diagnostic practice. Emphasize the limitations of partial recordings, which are not sufficient for comprehensive diagnosis and often lead to underdiagnosis.

### Uniform application of AAP/EFP classification

Encourage the consistent use of the AAP/EFP classification system during periodontal clinical examinations. This practice will ensure accurate staging and grading of periodontitis, thus enabling the development of individualized treatment plans.

### Enhance understanding of complementary diagnostic tests

Ensure dental professionals are thoroughly trained in the variety of diagnostic tests available for periodontal diseases, including imaging and laboratory tests. Focus on enhancing the effective application of these tests to improve diagnostic accuracy, according to the existing evidence about these tests.

### Support research on innovative diagnostic methods

Advocate for ongoing research into innovative methods for periodontal disease diagnosis that offer accuracy and less invasiveness. Promote the adoption of these emerging technologies and tools in clinical practice upon their validation.

### Comprehensive interviews and multidisciplinary collaboration

Provide dental practitioners with training on conducting in-depth interviews to explore systemic and behavioral risk factors for periodontal diseases. Encourage a multidisciplinary approach to care, by integrating insights from various health specialties into care

## Strategies for the Prevention of Periodontal Disease and its Impact on General Health

### Launch multifaceted awareness programs

Execute comprehensive programs across community settings such as schools, workplaces, and public spaces, using both traditional and digital media to highlight the risks and systemic health impacts of periodontal disease. Engage local influencers to extend the campaign reach and effectiveness.

### Develop and Implement Evidence-based Guidelines (EBG)

Formulate EBG to advocate for periodontal health across all life stages, emphasizing risk management and adherence to lifestyle, according to the characteristics of the LAC countries. Involve a broad spectrum of stakeholders in EBG development and ensure accessibility by providing materials in various languages and formats suitable for all literacy levels.

### Train dental professionals in patient communication

Equip dental professionals with the skills to effectively communicate the connection between oral health and overall wellness. Transform dental office waiting areas into educational hubs with resources such as videos, brochures, and interactive tools that promote healthy lifestyle choices.

### Support research on innovative health promotion methods

Invest in studies exploring the impact of mobile health applications, tele-dentistry, and social media on oral health awareness and self-care. Focus on assessing how these technologies improve access to dental care by marginalized communities.

### Incorporate oral health education into community programs

Integrate oral health education into schools and community initiatives, offering practical demonstrations on effective oral hygiene practices. Organize health fairs providing free dental screenings, and educational workshops to emphasize the significance of oral hygiene.

### Partner with dental product companies for resource distribution

Collaborate with companies to provide free or affordable oral hygiene products to populations in underprivileged areas. Advocate for the inclusion of oral health products in basic healthcare provisions and ensure the availability of products beneficial to managing advanced periodontal conditions.

### Emphasize personalized oral hygiene education

Stress the importance of tailored oral hygiene instructions in professional development sessions for dental practitioners, catering to the diverse needs of patients including those with special needs.

### Encourage longitudinal studies on the effectiveness of oral health strategies

Promote long-term research to evaluate the sustained impact of mechanical and chemical plaque control strategies. Focus on developing and testing oral hygiene products that cater to the region’s diverse population and investigating the role of prebiotic diet and probiotics in periodontal health.

### Develop integrated care programs for systemic and oral health

Create care models that integrate oral health assessments^13^ into routine check-ups for individuals with chronic diseases or during pregnancy. Offer training on the systemic implications of periodontal health for non-dental healthcare providers.

### Foster a network of professionals with periodontal disease expertise

Establish a collaboration network among dental and medical professionals to share knowledge, research findings, and educational resources on periodontal and systemic health interrelations.

### Build partnerships with patient associations

Work with patient organizations to spread information about periodontal disease prevention and its connection with systemic health, customizing educational materials for the needs of different communities.

### Support interdisciplinary research on preventive strategies

Back multicenter studies examining the effectiveness of periodontal disease prevention measures and their impact on systemic health, with the aim of disseminating information about comprehensive public health policies and clinical guidelines.

## Treatment of Periodontal Diseases

### Integrate comprehensive care into treatment plans

Incorporate a holistic care model into periodontal treatment that combines risk factor management with a mix of non-surgical and surgical interventions. Ensure a strong emphasis on Supportive Periodontal Care (SPC) to maintain oral health and prevent the recurrence of disease.^14^

### Strengthen patient engagement in treatment

Boost patient involvement in their periodontal health by focusing on biofilm control and mitigating risk factors. Promote a partnership between patients and dental professionals to achieve and sustain periodontal health, using patient education and motivation strategies that are culturally appropriate and multidisciplinary.

### Incorporate adjunctive therapies wisely

Encourage healthcare providers to judiciously integrate adjunctive therapies into periodontal care in accordance with the available evidence. Recommend the careful selection of adjunct treatments such as local antiseptics and antibiotics^15,16^ as supplements to mechanical plaque control and subgingival instrumentation when conventional methods are insufficient.

### Tailor Supportive Periodontal Care (SPC) programs

Develop personalized SPC programs^17^ that consider the connection between oral and systemic health and focus on enhancing patient adherence. Adjust these programs to meet the diverse cultural, socioeconomic, and literacy levels of the population, ensuring they are accessible and relevant to patients.

### Update dental education and practice

Advocate for the modernization of dental education and practice across Latin America and the Caribbean to align with contemporary, evidence-based periodontal care approaches. Emphasize affordable and practical interventions that cater to the regional socioeconomic conditions, and foster continuous professional development for dental practitioners.

### Advocate for comprehensive public health policies

Push for the development and enhancement of inclusive public health policies that address periodontal disease as part of the wider oral and general health agenda. These policies should focus on preventive care, timely intervention, and the integration of periodontal health services into the primary healthcare framework, enhancing access for marginalized communities.

## Conclusion

In summary, the 2024 Consensus on Periodontology articulates a comprehensive strategic approach, designed to tackle the significant public health challenge posed by periodontal diseases in the region. It simultaneously tackles critical areas such as disease prevalence, effects on oral health-related quality of life, systemic health correlations, and risk factors simultaneously with diagnostic, preventive, and therapeutic measures based on clear, actionable strategies. Thus the consensus lays a solid foundation for significant improvements in periodontal health across the region. Moreover, it aims to mobilize clinical and policy decision-making processes, ensuring that these strategies are effectively implemented. Their successful execution depends on the persistent, concerted efforts of dental practitioners, researchers, educators, and policy makers, all working in unison towards the common goal of ensuring all individuals within the region have equitable access to effective periodontal prevention, diagnosis, and treatment.
